# The European medical information framework: A novel ecosystem for sharing healthcare data across Europe

**DOI:** 10.1002/lrh2.10214

**Published:** 2019-12-25

**Authors:** Simon Lovestone, EMIF Consortium

**Affiliations:** ^1^ Neurodegeneration, Janssen R&D, Janssen Pharmaceutica, Beerse, Belgium

**Keywords:** catalogue, EMIF, EMIF‐AD, EMIF‐MET, use case

## Abstract

**Introduction:**

The European medical information framework (EMIF) was an Innovative Medicines Initiative project jointly supported by the European Union and the European Federation of Pharmaceutical Industries and Associations, that generated a common technology and governance framework to identify, assess and (re)use healthcare data, to facilitate real‐world data research. The objectives of EMIF included providing a unified platform to support a wide range of studies within two verification programmes—Alzheimer's disease (EMIF‐AD), and metabolic consequences of obesity (EMIF‐MET).

**Methods:**

The EMIF platform was built around two main data‐types: electronic health record data and research cohort data, and the platform architecture composed of a set of tools designed to enable data discovery and characterisation. This included the EMIF catalogue, which allowed users to find relevant data sources, including the data‐types collected. Data harmonisation via a common data model were central to the project especially for population data sources. EMIF also developed an ethical code of practice to ensure data protection, patient confidentiality and compliance with the European Data Protection Directive, and GDPR.

**Results:**

Currently 18 population‐based disease agnostic and 60 cohort‐based Alzheimer's data partners from across 14 countries are contained within the catalogue, and this will continue to expand. The work conducted in EMIF‐AD and EMIF‐MET includes standardizing cohorts, summarising baseline characteristics of patients, developing diagnostic algorithms, epidemiological studies, identifying and validating novel biomarkers and selecting potential patient samples for pharmacological intervention.

**Conclusions:**

EMIF was designed to provide a sustainable model as demonstrated by the sustainability plans for EMIF‐AD. Although network‐wide studies using EMIF were not conducted during this project to evaluate its sustainability, learning from EMIF will be used in the follow‐on IMI‐2 project, European Health Data and Evidence Network (EHDEN). Furthermore, EMIF has facilitated collaborations between partners and continues to promote a wider adoption of principles, technology and architecture through some of its continued work.

## 1 EMIF—INTRODUCTION

In recent years, the scientific research community has witnessed a surge in the form and amount of medical data which can be utilised for research purposes. There are currently hundreds of databases in Europe that store data from millions of patients. However, these data are scattered across multiple platforms, each with its own legislations and guidelines, which limits their use.^1^, ^2^ Standardizing these data to a common platform would provide uniform representation and architecture thereby enabling the standardisation of data and allowing the use of common analytical tools.^3^ This would maximise the research value of the scientific information and allow researchers to make significant advancements in medical research and drug development leading to improved quality of care through data discovery and analysis.^4^ Therefore, there is a need for a unified platform that allows researchers to find, assess and (re)use healthcare data on a wider scale, bringing together data from different populations. This would allow researchers to increase sample sizes and to run parallel analyses in different countries which would otherwise be challenging using multiple databases on diverse platforms.^1^, ^3^ The development of the European medical information framework (EMIF) was one such initiative that provided researchers with a federated network and an analysis platform enabling the reuse of large‐scale harmonised patient data in a structured way. It was a complex project combining technology, ethics and health research.^5^ The goal of EMIF to increase access to human health data were achieved via a three‐phase approach: (a) by assimilating data source profiling information and allowing *bona‐fide* researchers (an individual irrespective of its affiliation, interested in conducting research for the common good and who does not investigate data from a marketing or sales perspective) to browse metadata; (b) by providing a single point of access for searching aggregated data across different sources and countries; and (c) by enabling *bona‐fide* researchers to answer specific questions such as identifying and validating novel biomarkers through advanced data analysis. With a foundation in an open science and open source culture, EMIF facilitated collaborations between diverse data custodians, academics, subject matter experts, patient organizations and 10 pharmaceutical companies.

The aim of EMIF was to develop a common information framework of patient‐level data that would standardize and facilitate access to diverse medical and research data sources, thereby allowing new research avenues to be explored. To achieve this, a common framework, governance and technology platform, called EMIF‐Platform (EMIF‐PLAT) was developed to find, assess and (re)use the health data from diverse sources across Europe. In addition to the technological developments, an EMIF code of practice (ECoP) was developed to provide an ethical governance framework, ensuring that the EMIF‐PLAT and services were compliant with the respective national guidelines, European Data Protection Directive, and now General Data Protection Regulation (GDPR). Furthermore, the ECoP also provides specifications on how the EMIF project and future users of EMIF may comply with the Innovative Medicines Initiative project (IMI) code of practice.

The EMIF‐PLAT was built around two main data‐types: electronic health record (EHR) data and cohort data; data sources that are very different in nature and structure. Cohort data most often contains deep phenotypic characterization of research participants, sometimes including rich biomarker data, and consequently, cohort databases have fewer subjects than the EHR databases but may have higher demands for harmonization. Both data‐types need specialised tools to find, evaluate and analyse the respective data sets. The EMIF catalogue contains high‐level metadata and is the starting point for researchers seeking to access EMIF data in EMIF‐PLAT. Additionally, other modules were developed that allowed documentation, task and research process management and analysis of the requested data in a secure environment. All access controls were handled by data custodians. Researchers can request access to the EMIF Catalogue, join specific communities of their interest, search and explore sets of detailed meta‐information, assess data suitability and the feasibility of a particular study in selected databases and finally, after approval of the data owners(s), conduct the proposed study in a secure data analysis environment.

Since its inception in 2013, EMIF has accomplished multiple deliverables while simultaneously overcoming considerable challenges. The current manuscript emphasizes the role played by EMIF in performing multi‐database studies across Europe in a harmonised, standardised and efficient way. Additionally, it provides an overview of the underlying technology, key outputs and the challenges encountered during the establishment of the project and achieving the desired outcomes. Further, the manuscript highlights the role of EMIF in providing a learning foundation for the future projects.

## 2 OBJECTIVES OF EMIF

The EMIF project was structured around three main research objectives: (a) the establishment of the information framework (EMIF‐PLAT) for evaluating, enhancing and providing access to human health data across Europe and support EMIF‐AD and EMIF‐MET, (b) identification of predictors and diagnostic and prognostic biomarkers of AD (EMIF‐AD), and (c) identification of predictors and diagnostic markers of metabolic complications of obesity in adults and children (EMIF‐MET).

### 2.1 EMIF‐Platform

The primary objective of EMIF‐PLAT was to facilitate the (re)use of healthcare data. To this end, EMIF developed different modular components to support identification, assessment and analysis of the information together on a common platform. This platform allowed researchers to find the specific data sources that meet their requirements, provided data visualisation options and supported the workflow and cooperative work between researchers and database owners.

### 2.2 EMIF‐AD

EMIF‐AD was set‐up to facilitate the process of combining and reusing existing data, aiming at the discovery of new diagnostic and prognostic biomarkers for AD. Further, it also provided use cases which shaped the requirements for the platform and served as a validation of developed applications. Besides allowing researchers to browse cohort metadata in the EMIF‐AD Catalogue, a web‐based browser was developed to allow search queries on harmonised cohort data in order to identify cohorts holding relevant information for specific research questions. A user with a valid access was allowed to explore the information. In addition to allowing the reuse of AD cohort data, EMIF‐AD also set up the EMIF‐AD Multimodal Biomarker discovery (EMIF‐AD MBD) study.^6^ Using these samples, the available data on each subject was then enriched with multi‐omics (genomics, metabolomics and proteomics) data. In this study, virtual cohort of 1221 participants selected across 11 AD dementia research cohorts with the criteria for inclusion being the (a) age > 50 years and (b), the availability of an amyloid status (measured in CSF or by amyloid‐PET). While metabolomics and proteomic analyses were performed in plasma and CSF, genome‐wide single nucleotide polymorphism genotyping and next‐generation sequencing were conducted in DNA samples.^6^ To facilitate data sharing and analysis in the EMIF biomarker discovery study, a database taxonomy based on the AD metadata elements (fingerprints) and data dictionary was established. All data were harmonised to this newly established data set and thereafter stored in common platform tranSMART, which was used as an integration platform.^7^ By using existing data supplemented with the novel data from existing samples, EMIF‐AD was able to establish a very large cohort of research participants for biomarker study at relatively low cost and relatively high speed demonstrating the efficiency savings of (re)use of data.

### 2.3 EMIF‐MET

EMIF‐Metabolic aimed to identify risk markers for metabolic complications of obesity. Obesity is a heterogeneous condition in which many obese individuals do not show evidence of the metabolic complications of obesity and, conversely, many non‐obese individuals show a dysmetabolic state. The Metabolic topic focused on identifying biomarkers of risk and mechanisms related to this heterogeneity and tested these biomarkers in small and medium‐sized cohorts followed by testing in large clinical populations with outcome data. Identifying useful biomarkers of obesity‐related complications (Type 2 diabetes [T2D], cardiovascular disease [CVD], cancer and non‐alcoholic fatty liver disease [NAFLD]/non‐alcoholic steatohepatitis [NASH]) could facilitate more efficient and focused clinical trials and influence the risk‐benefit balance for novel therapeutics by targeting treatments to those at highest risk.

## 3 EMIF ARCHITECTURE AND KEY OUTPUTS

### 3.1 EMIF‐Platform

The EMIF‐PLAT was based on a federation of data sources, rather than a centralised database containing all data. Therefore, the tools developed were capable of running locally at the data custodian site, preserving local provenance and governance, providing data security by design. These tools performed anonymisation and study‐specific data extraction, and helped in the identification, assessment and (re)use of health data.

**Figure 1:**
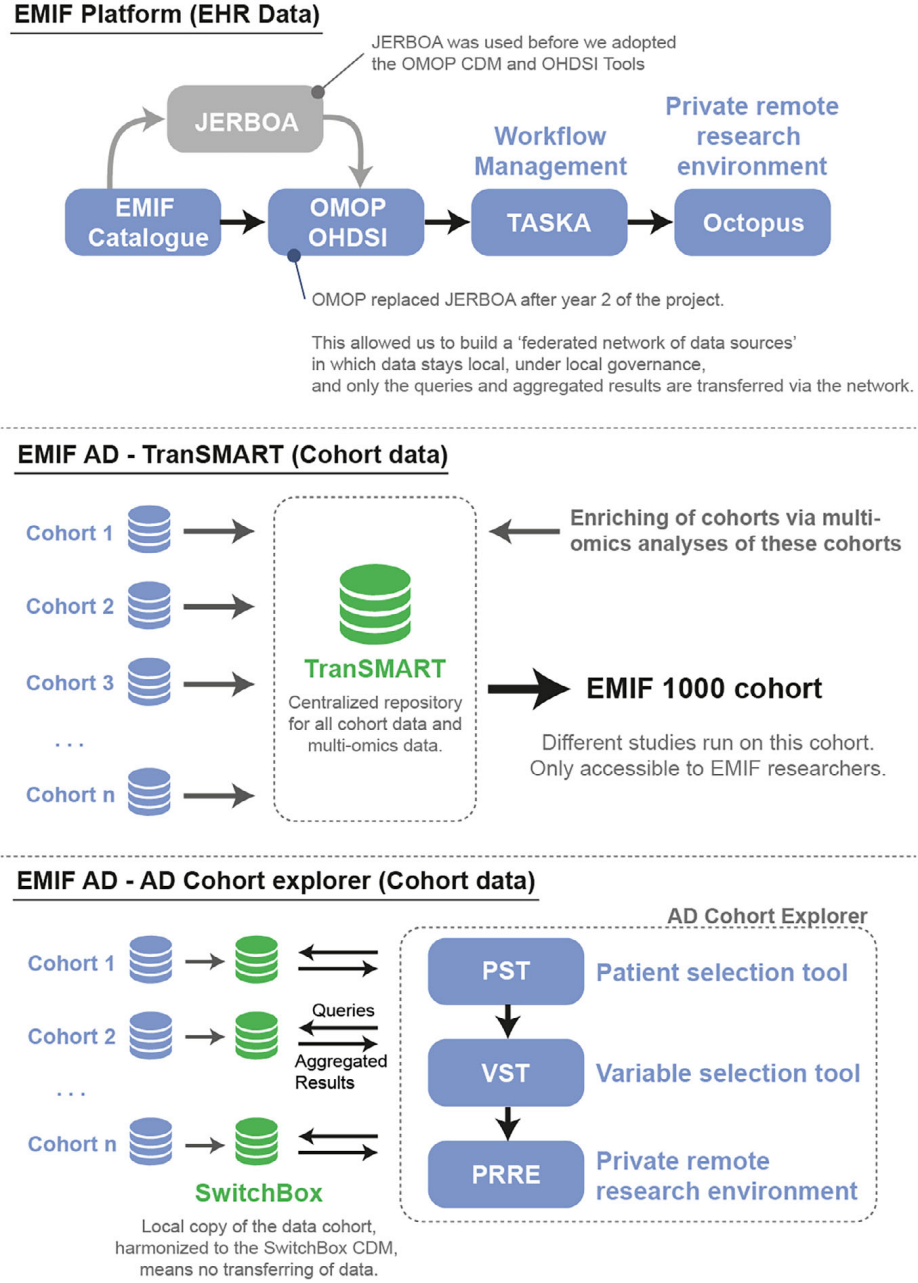
Schematic overview of the infrastructure built during the EMIF project. **EMIF Platform** has built a federated network of data sources. While JERBOA was initially used, EMIF later switched to using the OMOP CDM and OHDSI tools which were not available at the project commencement. In doing so, a federated network of data sources harmonized to the OMOP CDM was built, on which studies could be run using TASKA as a workflow management tool and Octopus as a private remote research environment. **EMIF AD,** on the other hand, used two separate infrastructures. The first one relied on TranSMART as a central data repository in which data from cohorts were stored and enriched via multi‐omics analysis on samples of these databases. This allowed us to build the EMIF 1000 cohort which was used to run the EMIF studies. The second infrastructure, however, was the EMIF AD cohort explorer. In this setup, AD cohorts harmonized their data to the Switchbox (an AD specific common data model based on the OMOP CDM). The AD Cohort explorer could then send queries to these harmonized databases after which the aggregated results were shown in the AD cohort explorer (consisting of the patient selection tool [PST] and variable selection tool [VST]). If the cohort custodian agreed to run a research study, the requested data would then be made available in the private remote research environment (PRRE) of the AD cohort explorer, thus implying that both data and governance once again remain local

The architecture of EMIF had to support two different data‐types, EHR data and cohort data (Figure 1). It also took into consideration different requirements for each of the data‐types such as harmonization and privacy protection. The biggest advantage of this dual approach, however, was that it allowed EMIF to create appropriate tools to address specific problems or research goals in both tracks. Several shared tools were leveraged to support both data‐types. For example, the EMIF catalogue served as an entry point for both sources to the EMIF‐PLAT. Among the other tools were the Jerboa tool that enabled federated analyses of the EHR data sources, the patient selection tool (PST), the variable selection tool (VST), TASKA (research task management tool), private remote research environments (PRRE), Codemapper (developed under the aegis of IMI ADVANCE project), integration of ATLAS tool from observational health data sciences and informatics (OHDSI), These tools are discussed more in detail below.

**Figure 2:**
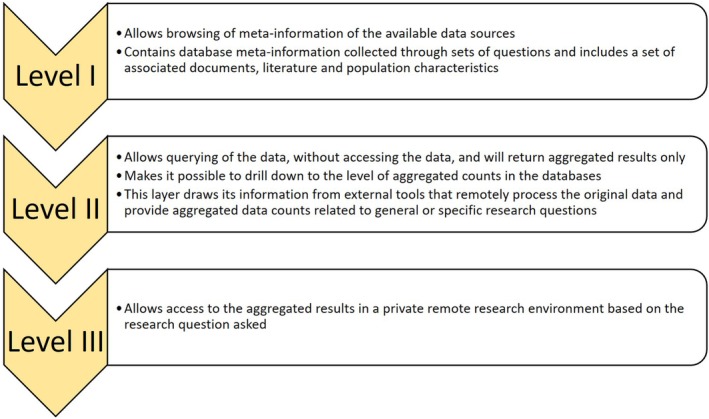
Different levels of access to data sources

The EMIF Catalogue is the main component of the EMIF‐PLAT and it allows integration of a large range of biomedical metadata coming from multiple institutions, using a common metadata schema—fingerprint—for each data type (eg, EHR repositories, disease‐specific cohorts, etc.). Currently, 18 population‐based disease agnostic and 60 cohort‐based data partners from across 14 countries collaborating within EMIF are contained within the catalogue. The catalogue allows evaluation of data source suitability and initiate new research studies with the data sources in a secure environment. Data custodians have full control on data visibility levels, thereby ensuring fine‐grained access control to the metadata. The EMIF Catalogue (https://emif-catalogue.eu) was developed over the Montra open source platform^8^ and handles the data collected from different EHR databases. It is designed to be compatible with the Jerboa output formats. Several levels of information are accessible in a controlled and remote environment. A role‐based access control system is also deployed to enforce EMIF access policies, which could be tailored to combine these access rules with researchers' privileges. Using these perspectives, an approach that would allow access to data sources at different levels of detail, while maintaining data privacy is possible (Figure 2). By promoting the data publishing, data discovery and data (re)use, the EMIF catalogue allows researchers to identify databases of interest, thereby helping to conduct studies while reducing the overall time and resources required for the completion of studies using healthcare data.^9^

To enable federated study execution on the EHR database, the EMIF project invested considerable effort in improving a Java tool called Jerboa reloaded. This tool was initially developed within the exploring and understanding adverse drug reactions (EU‐ADR) project, and subsequently applied in numerous other projects.^10^ The tool was re‐engineered in EMIF and functionally extended considerably, for example, to enable new study designs, include graphical feedback to the data custodian, and add quality control functionality. The EMIF project has made the tool opensource so others can contribute to the functionality and apply the tool for their own studies (https://github.com/mi-erasmusmc/Jerboa). The software runs against common input files to harmonise the data elements needed for a specific study. It then carried out de‐identification and executed a study design as specified in a script. The big advantage of the Jerboa framework was that the same analysis was run on each database, thereby eliminating possible implementation bias from the local statisticians. Jerboa employed a flexible, modular design approach, that is, the custom‐built script language allowed to combine modules into a full study. For example, a script could start with a population definition, then an advanced cohort definition, have an outlier detection model, and then apply a case control design. The final result of a local Jerboa run was an analytical data set that could be shared with the study team, for example, through the PRRE. A simple example is the profiling data in the EMIF Catalogue or the EHR databases, which was generated by Jerboa and then pushed to the webtool.

Different layers of the EMIF architecture supported data discovery through community‐based catalogues, dashboard functionality, database querying and tools that allow central analysis in a private remote research environment. Several freely available OHDSI tools, such as ATLAS, were integrated and new features developed. The latter greatly expanded the data assessment and analytical capabilities of the EMIF Catalogue.^11^, ^12^ Another asset adopted from the OHDSI community is the observational medical outcomes partnership (OMOP) common data model (CDM), used to standardise the format and content of observational data so that common software applications, tools and methods could be applied across data sets from multiple healthcare organizations thereby allowing faster analysis across a huge number of federated data sets. While JERBOA was initially used, EMIF later switched to using the OMOP CDM and OHDSI tools which were not available at the project commencement. In doing so, a federated network of data sources harmonized to the OMOP CDM was built, on which studies could be run using TASKA as a workflow management tool and Octopus as a private remote research environment (illustrated in Figure 1).

**Figure 3:**
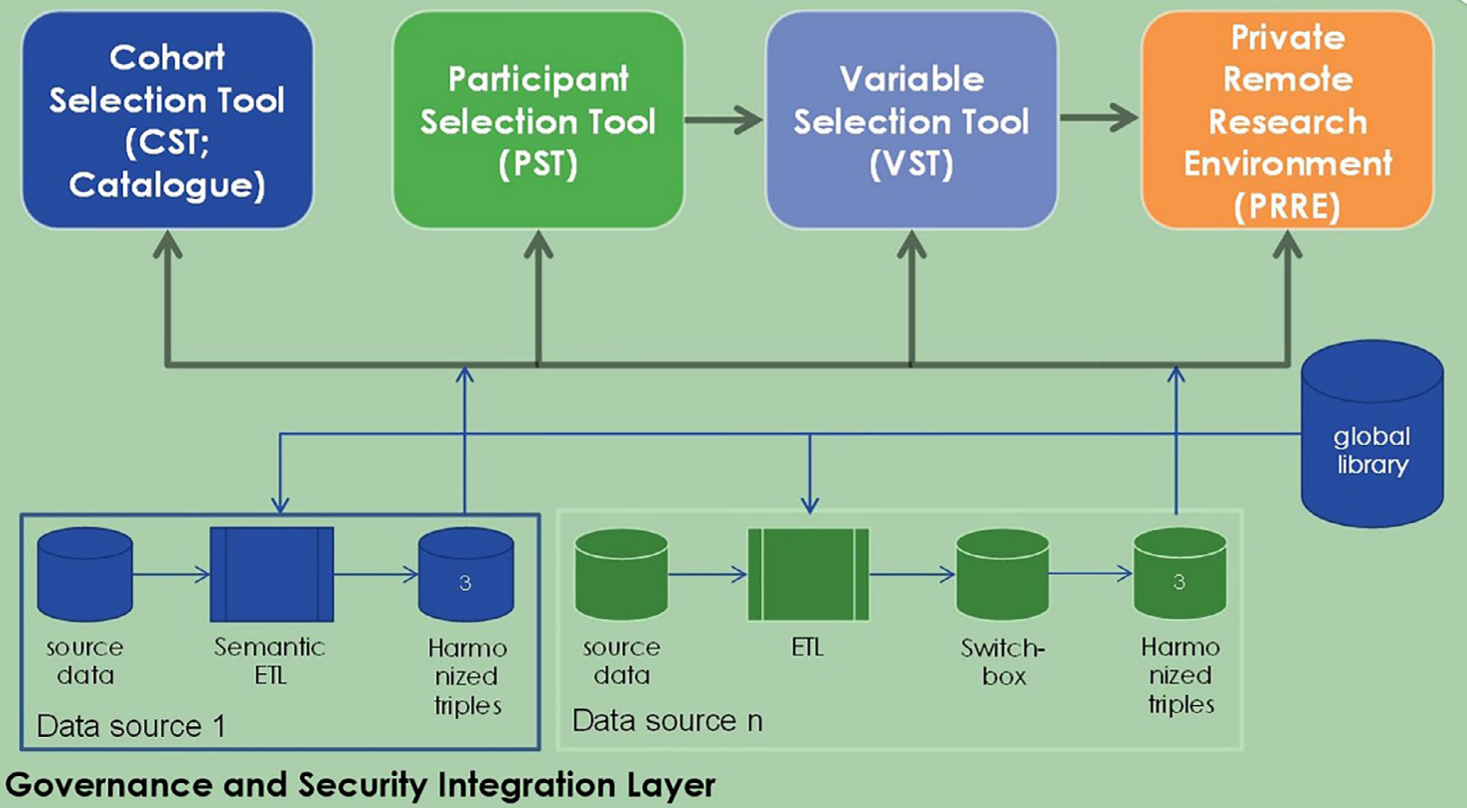
A high level overview of cohort architecture. **Components of cohort architecture**: *CST (cohort selection tool)*: This tool provides the researcher with an overview of the availability and applicability of potential cohort data. *PST (participant selection tool)*: The PST allows a researcher to get an overview of patient profiles in a given cohort (currently only supports the AD cohort data sets), filtered on a set of predefined key characteristics. *VST (variable selection tool)*: The VST allows a researcher to get an overview of available variables (aggregated counts, not the actual values). This is followed by a request to the selected cohort owners for data access. *PRRE (private remote research environment)*: For EMIF‐AD, a secured data platform called TranSMART is used for storing, managing and analysing all cohort data. Data uploaded to tranSMART is anonymised and harmonised according to the EMIF‐AD common data model to enable pooling of different cohort data

On the parallel EMIF‐AD cohort architecture, EMIF‐PLAT supported data discovery and (re)use workflow through the cohort selection tool (CST or Catalogue), PST, VST and PRRE (Figure 3). Thus, although these two tracks followed the same workflow, they had parallel implementations. While the CST provided researchers with an overview of the potential EHR data as well as cohort data, availability and suitability, the PST was meant to identify subgroups in cohorts, filtering on a set of predefined key characteristics, that satisfy inclusion or exclusion criteria for a study thereby allowing researchers to set up a virtual cohort for further analysis. The VST provided researchers with an overview of the available variables (counts, not values), followed by the possibility of submitting a data access request to the selected cohort owners.

### 3.2 Data harmonisation

EMIF managed to develop a common architecture for both population and cohort data by incorporating specific harmonisation approaches via collaboration with the international OHDSI community and adoption of the OMOP CDM^12^ for population data. The adoption of OMOP CDM was a core tenet for federation. EMIF has mapped nine European population data sources to the OMOP CDM and OHDSI vocabularies: Denmark (AUH),^13^ Italy (ARS),^14^ IMS HEALTH LPD,^15^ PEDIANET,^16^ Spain (IMASIS),^17^ SIDIAP,^18^ UK (THIN),^19^ Estonia (EGCUT)^20^ and the Netherlands (IPCI).^21^

Although, the team was not in a position to run federated queries during the EMIF project, this is now being attempted at a large scale within the follow‐on IMI2 European Health Data and Evidence Network (EHDEN) project. A common ETL (Extract, Transform, Locate) team conducted the mapping to the OMOP CDM, thereby reducing inconsistencies between individual data custodians, who for the most part did not map to the CDM themselves, while also ensuring that mapping was performed collectively by a group of data custodians. Importantly, working closely with each data custodian prior to, and during the mapping cycle is considered extremely important in addressing any contextual issues, especially if there are inconsistencies, missing data or completeness issues.

Within a federated model, the governance requirements, including consent, stay local, meaning that running queries is wholly dependent on this being allowable by local approval, not simply because data have been mapped to the OMOP CDM.

Compared to population‐based data sets, patients in research cohorts were deeply phenotyped, often using disease‐specific evaluations, imposing high levels of semantic compatibility.

The EMIF harmonisation framework for cohorts mapped data source variables to a pre‐defined template of variables. During harmonisation of data to this template, involvement of the data custodians was required due to their expertise in the protocols used to collect the data. A common framework, called knowledge objects, based on semantic web technology captured the data as well as the protocol or variable descriptions in a structured way. Rules defined the (possibly complex) transformations between knowledge objects, generating tree‐like dependencies from an analysis variable up to the source measurements, allowing full provenance tracking. In a geographically diverse environment such as EMIF, the knowledge object framework was also used to assign the security levels to users and to build a library of reusable objects between data analysis projects.^22^ The knowledge object's internal data structure was sufficiently general to accommodate all clinical data‐types anticipated in the EMIF verticals. Public vocabularies were used whenever possible to annotate relevant aspects of a measurement protocol.

EMIF developed an in house minimal data set for AD cohort data called as Switchbox, which was a common data model of 241 harmonised variables relevant to AD research, derived from the OMOP common data model.^23^ The Switchbox allows users to query the harmonised AD data cohorts at a group level, via the included PST and VST.

### 3.3 TranSMART for EMIF‐AD

To meet the need of storing large amounts of harmonised data in EMIF‐AD, an ad‐hoc process was set up for cohort onboarding, data harmonisation and upload into tranSMART. The latter allows accessing the data besides offering the search and analysis capabilities, thereby offering to develop and refine the hypothesis of research questions.^24^ The software is based on the international standard, i2b2, clinical data warehouse model comprising an entity attribute value pair‐derived star‐schema.^24^, ^25^ It handles data from clinical trials and biomarkers including gene expression profiles, genotypes, proteomics and metabolomics.^25^, ^26^ In addition, only when required and if agreed to by data custodians, the data from tranSMART could be accessed or exported to SAS, R or MS Excel software in case further analysis was warranted from statisticians.

All cohort data uploaded to TranSMART was deidentified beforehand, either at the source or by a trusted third party (TTP, Custodix) and stored on a secure OwnCloud server (https://owncloud.org/) at Custodix. Data loading procedures in TranSMART required that data sets, for which data owners were unsure of de‐identification requirements, be de‐identified by Custodix before they could be made available to the TranSMART team for data upload. A library of harmonised variables was maintained using Webprotégé.^27^ Variables ranged from demographics and subject characteristics, clinical information and lab tests over AD‐specific measurements (cognitive screening, rating scales and neuropsychological examinations) to imaging and pharmacogenetic findings. For each cohort, a custom script was developed to transform the source variables into harmonised variables. The custom‐scripts were used as an input to develop a more sustainable architecture described above (knowledge objects and Switchbox). The script and harmonised data set were uploaded to OwnCloud. The EMIF‐PLAT team developed tools that connected to OwnCloud for data and to Webprotégé for the corresponding variable taxonomy and uploaded the data to tranSMART. The tranSMART server was hosted at the TTP. Access to the data could be requested for specific EMIF‐AD tasks or deliverables and was assessed on a case‐by‐case basis.

### 3.4 Use case development

The EMIF project aimed to address scientific questions in the field of AD and obesity, the so‐called use cases (Table 1), while at the same time testing the capabilities of EMIF data sources and driving the development of the EMIF‐PLAT tools and processes. Each use case had its own research team, responsible for the correct and timely execution of the use case. This research team typically consisted of clinical experts, epidemiologists and data analysts as well as data custodians including members from both academia and industry were equally involved. This collaboration between academic and industry partners also helped to increase trust between the different stakeholders. Some of these use cases have been published.^28^, ^29^ Although use cases focused primarily on AD and MET, they were also instrumental in covering the broader epidemiological study designs ranging from the estimation of incidence and prevalence of diseases and associated comorbidities to the determination of treatment patterns in patients, using different databases.

**Table 1:** List of use cases

Use case	Title
1	Dementia prevalence and incidence in a federation of European Electronic Health Record (EHR) databases.^29^
2	BMI and the risk of cardiovascular disease and all‐cause mortality in European electronic medical records databases.
3	Association of non‐alcoholic fatty liver disease with cardiovascular and liver morbidity in electronic health record databases^28^
4	Dementia: vascular and metabolic risk factors.
5	Treatment pathway analysis: An evaluation of treatment patterns and drug utilisation among cases with incident dementia in EHR databases available in the European Medical Information Framework (EMIF).
6	A nested case–control study of prior history of non‐alcoholic fatty liver disease in demented and cognitively impaired individuals matched to healthy controls in European health records data.
7	Utilisation of healthcare data to identify sub‐types of heart failure patients based on clinical and/or molecular phenotypes
8	An exploratory phenome wide association study linking asthma and liver disease single nucleotide polymorphisms and electronic health records from the Estonian Genome Centre at the University of Tartu Database^61^
9	Investigating the relationship in paediatric population between antibiotics dosing of antibiotics (prescribed, dispensed or administered) and patient's weight.
10	Trazodone and the risk of dementia: an electronic primary care records analysis.
11	Identifying cases of type 2 diabetes in heterogeneous data sources: strategy from the EMIF project^62^

*Note:* The EMIF‐AD program sought to generate a platform to enable efficient reutilisation of pre‐existing data. Table 1 lists the project use‐cases for reutilisation of this data proposed as the program was set up. Three of these were completed with papers generated as referenced (see below) and others are in various phases of development. However, in addition to these use‐cases, EMIF‐AD had one large “meta use‐case” to re‐use existing cohort data to identify participants to studies who had generated data and donated biofluid samples that would enable biomarker discovery and validation studies. Specifically, we sought to identify biomarkers to facilitate therapeutic trials. This use‐case was singularly successful, rapidly generating a virtual cohort assembled from pre‐existing cohort data and then accessing samples from these individuals. This process is described in Bos et al (Bos I, Vos S, Verhey F, et al. Cerebrospinal fluid biomarkers of neurodegeneration, synaptic integrity, and astroglial activation across the clinical Alzheimer's disease spectrum. Alzheimer's dement. 2019;15(5):644‐54.) and some of the published outcomes listed are here (van Maurik IS, Vos SJ, Bos I, et al. Biomarker‐based prognosis for people with mild cognitive impairment (ABIDE): a modelling study. Lancet Neurol. 2019. https://doi.org/10.1016/S1474-4422(19)30283‐2; Shi L, Westwood S, Baird AL, et al. Discovery and validation of plasma proteomic biomarkers relating to brain amyloid burden by SOMAscan assay. Alzheimer's Dement. 2019. https://doi.org/10.1016/j.jalz.2019.06.4951; Morgan AR, Touchard S, Leckey C, et al. Inflammatory biomarkers in Alzheimer's disease plasma. Alzheimer's dement. 2019;15(6):776‐787; Kim M, Snowden S, Suvitaival T, et al. Primary fatty amides in plasma associated with brain amyloid burden, hippocampal volume, and memory in the European Medical Information Framework for Alzheimer's Disease biomarker discovery cohort. Alzheimer's dement. 2019;15(6):817‐27; Westwood S, Baird AL, Hye A, et al. Plasma protein biomarkers for the prediction of CSF amyloid and Tau and [18F]‐Flutemetamol PET scan result. Front Aging Neurosci. 2018;10:409; Ten Kate M, Redolfi A, Peira E, et al. MRI predictors of amyloid pathology: results from the EMIF‐AD Multimodal Biomarker Discovery study. Alzheimers Res Ther. 2018;10(1):100; Bos I, Vos S, Vandenberghe R, et al. The EMIF‐AD Multimodal Biomarker Discovery study: design, methods and cohort characteristics. Alzheimers Res Ther. 2018;10(1):64; Hong S, Prokopenko D, Dobricic V et al. Genome‐wide association study of Alzheimer's disease CSF biomarkers in the EMIF‐AD Multimodal Biomarker Discovery dataset. bioRxiv. https://doi.org/10.1101/774554.) with others in generation (Hong S, Prokopenko D, Dobricic V et al. Genome‐wide association study of Alzheimer's disease CSF biomarkers in the EMIF‐AD Multimodal Biomarker Discovery dataset. bioRxiv. https://doi.org/10.1101/774554.).

## 4 KEY OUTPUTS OF EMIF‐AD AND EMIF‐MET

### 4.1 EMIF‐AD: Early biomarker development and disease insights

#### 4.1.1 Early biomarker development

Treatments for AD developed during the last two decades have largely failed to ameliorate the disease course or to alleviate symptoms over a longer period of time, despite significant contributions by governments, industry and private donors.^30^ This has led to a shift in strategy, focussing on treating patients earlier in the disease course. Therefore, considerable work is currently being conducted on developing biomarkers for the identification of AD at an earlier stage, especially low invasive biomarkers such as those in blood.^7^, ^30^ The EMIF‐AD project attempted to identify potential biomarker targets by making optimal use of the plethora of readily available AD cohort databases for research, including biomarker discovery, a topic that was used as an example programme.

#### 4.1.2 Disease insights

Several research questions were put forward by EMIF‐AD including that of aiming to identify a reliable biomarker. Importantly, by reusing the already available AD patient data, these studies have helped to gain deeper insights into disease pathophysiology. The EMIF‐PLAT fulfilled its stated objectives with several outputs of its research projects now published in peer‐review medical journals.^6^, ^31^, ^32^, ^33^, ^34^ Taken together, these studies show that EMIF‐AD has advanced the AD biomarker field significantly, and the developed tools are critical to allow data access at scale required for collaborative studies on people with a preclinical AD phenotype.

### 4.2 EMIF‐MET: Early biomarker development and disease insights

#### 4.2.1 Early biomarker development

EMIF‐MET aimed to identify the genetic causes of obesity and establish their potential relation with obesity complications (T2D, CVD and NAFLD/NASH). Further, it aimed to characterise the population and determine the biomarkers predicting the risk irrespective of the degree of obesity. Similar to EMIF‐AD, an analytical framework was constructed and data from ~20 000 obese patients was collected from existing medium‐sized genetic epidemiology cohort studies such as METSIM, EPIC‐Norfolk and Fenland.^35^, ^36^, ^37^

EMIF‐MET initially identified biomarkers associated with the risk of metabolic obesity complications. In well‐characterised small cohorts with extreme phenotypes and multiple omics data, followed by a comprehensive analysis of the data using appropriate tools to identify biomarkers of interest for further validation. These biomarkers were then validated and assessed for the causal significance in a well‐characterised medium‐sized cohort. The identified biomarkers were tested for their ability to predict the obesity‐associated outcomes using prospective and retrospective studies by selecting samples from the appropriate cohorts. The results were further compared with conventional predictors of disease outcome, such as risk factors, and demographic information. Consistent with these objectives, molecules related to insulin secretion capacity, insulin resistance and NAFLD were validated in a medium‐sized cohort.

#### 4.2.2 Disease insights

Numerous biomarker identification studies assisted by EMIF‐MET highlighted new findings to provide a platform to validate these biomarkers.^38^, ^39^, ^40^, ^41^, ^42^, ^43^, ^44^ Altogether, some important clinical questions in the real‐world data were addressed that until now, had mainly been investigated in small cohort studies, particularly in the NAFLD/NASH arena.

## 5 CHALLENGES IN THE PROJECT

### 5.1 Governance and provenance of data

EMIF aimed to establish a common information framework of patient health data by standardizing different medical data sources.^17^ This network would allow researchers and healthcare providers to identify, assess and reuse aggregated patient data, and most importantly, in a manner acceptable to all the stakeholders. The EMIF‐PLAT developed a Code of Practice (known as the ECoP) to ensure privacy protection of data subjects and to protect the interests of all data‐sharing parties. The goals in developing the ECoP were that the EMIF‐PLAT and its services are used in ways that comply with legislation and various organizational policies on data protection, that EMIF upholds best practices in the protection of personal privacy and information governance, and eventually that EMIF could promote best practices in the conduct of research using health data, for the general (public) interest.

EMIF needed to ensure compliance with Member State law based on the European Union Data Protection Directive. Given its recent enforcement, it was imperative for the ECoP to prepare for and comply with the new GDPR 2016/679. In developing the ECoP, several pre‐existing codes and policies were examined during 2014‐2015 to evaluate whether component parts of these should be adopted by EMIF. EMIF adheres to the IMI code on data privacy, which contains many high‐level principles that the ECoP could extend with more operational detail.^4^, ^45^, ^46^, ^47^, ^48^, ^49^, ^50^, ^51^, ^52^, ^53^, ^54^, ^55^ Successive drafts of the ECoP were developed during 2014‐2016, primarily by a core team of academic, healthcare, pharma industry and legal experts, with periodic wider consultation within the consortium comprising many data custodians, research users and patient organisations across Europe. A further layer of consultation occurred through presentations of the evolving work, and key issues at European and international conferences, and contributions to academic publications.^56^, ^57^

The ECoP specifies rules for the appropriate conduct of research users, data providers and intermediate brokers such as EMIF, when undertaking research using big and/or federated health data sets. It focuses on respectful data use, adhering to permitted uses, maintaining records of users and use, protection of data subject privacy, acknowledging data custodians, complying with governance rules and being subject to routine monitoring and investigational audit. It stipulates transparency by data custodians on the nature of their data subject population, the quality and currency of their data, of the arrangements for data sharing, including permitted uses, their approvals process, access fees, publication policy and governance requirements. Although predating the publication of findability, accessibility, interoperability and reusability (FAIR) principles, the ECoP aligns with these perfectly.

According to the ECoP, confirming ethical approval for data sharing remains the responsibility of the data custodian, including obtaining any additional approvals needed to cover any specific intended research use of the data. Both data custodians and research users are responsible for ensuring that any relevant participant consent meets the requirement for intended research. The ECoP deliberately does not stipulate the rules for scientific assessment, statistical validation and so forth, since these are addressed by other recognised data sharing codes of practice and templates (eg, some of those in the list given above).

The most important challenges the ECoP team faced were: how to define, control and monitor the purposes (kinds of research) for which federated health data are used; the kinds of organisations that should be permitted to conduct research; and how to monitor this.^4^ This ECoP will also be adopted in EHDEN, which intends to harmonise ~100 million EU records to the OMOP common data model, to facilitate—outcome‐based research.^58^ Details about ECoP and the challenges involved in ECoP have been explained in detail in a paper by Floridi et al.^4^

### 5.2 Agreeing on the terms of engagement around real‐world data

EMIF aimed to incorporate the benefits of RWD such as applications in biomarker discovery, predictive modelling, study design and post‐marketing drug surveillance. EMIF played a key role as a catalyst and provided a platform to explore the use of RWD within a federated network. One of the most important concerns was the need to adhere to local governance and provenance requirements. A balance had to be sought between these concerns and the tremendous need for data to be shared. Additionally, sufficient flexibility was required in terms of timelines, administrative burden and cost when conducting a research study. An example includes the EMIF‐AD MBD study in which a major proportion of the time (September 2014‐September 2016) of the total project duration (June 2014‐May 2018) had to be invested in contracting and negotiating the data sources. Furthermore, standardising different data sources in the OMOP common data model was a challenge owing to regulatory and governance restrictions. Moreover, the enormous diversity in clinical data presented a significant challenge to EMIF and its collaborators. One solution provided to this issue was the simplification of the data for integration and analysis. Importantly, with a foundation in an open science and open source culture and ensuring an agreement over evolving RWD terms, EMIF has an advantage over a closed market model that may pose hindrances with expectations, such as data sharing.

### 5.3 Simultaneous set up and application of platform

While EMIF was trying to establish a balance between the use of RWD and associated governance, developments such as technological advances via semantic tools, regulatory requirements (eg, GDPR), nature of RWD and research requirements continued to happen in the field of real‐world evidence. Therefore, it had to be ensured that the tools within EMIF were compliant whenever requirements were updated. For example, the collaboration with OHDSI and the adoption of the OMOP common data model were nascent at the beginning of EMIF, as were the number of tools used across the EMIF project. Similarly, as EMIF‐AD, EMIF‐MET and EMIF‐PLAT began at the same time, this required EMIF researchers to run studies on a platform that was simultaneously being set up, akin to ‘trying to fly a plane while you're still building it’.

### 5.4 Sustainability scenarios and operational model

From the get‐go, one of the goals of EMIF was to sustain the technology and governance framework for the use of data for medical research in the post‐IMI phase. The goal to have a meaningful impact on medical research in this post‐IMI phase was taken into the sustainability business model. During the final EMIF‐consortium meeting, EMIF acknowledged the efforts taken to develop a desired long‐term model and laid down a roadmap for the future work. Although network‐wide studies using EMIF were not conducted during this project to evaluate the sustainability of EMIF, it was imperative that the many lessons learned during the EMIF project are incorporated into currently ongoing and future IMI projects that focus on reusing healthcare data (eg, EHDEN). In addition, EMIF is currently also contributing to other projects, outside of the IMI framework, such as (but not limited to) the Dementias Platform UK (Home—Dementias Platform UK). EMIF helps these projects by making available the EMIF catalogue infrastructure and contributing to data harmonisation. At the same time, it is critical that the collaboration between all stakeholders continues within the developed EMIF framework and architecture to support RWD‐based research within Europe and internationally. This will help to improve its cost‐effectiveness and benefit the patients with respect to the development and access to and outcomes from therapeutic interventions. Interestingly, a study that evaluated several patient databases (16 projects of three funding agencies: USA's National Institutes of Health, European Commission and IMI), including EMIF, found that most of the projects (90%) already conduct, or plan on conducting, data maintenance, thereby making the data available for future use. It was further observed that more than half of the projects (65%) will continue to provide results to academics and some of the projects (20%) intended to develop a business model to sustain the research work on completion of the projects.^59^

Initial thinking during the development of EMIF focused on the potential for a legal and concrete entity as a one‐stop shop for RWD projects within a federated network. Additionally, leveraging the developed infrastructure into a self‐sustaining business is an enormous task, and the process is underway. This includes tasks such as scaling up, building on the OHDSI/OMOP common data model collaboration and a federated approach to European health research. Thus, a foundation has been laid for the follow‐on projects of EMIF (IMI2 initiated better data for better outcomes [BD4BO] programme and EHDEN project) and this will ensure that EMIF continues to promote a wider adoption of principles, technology and architecture, (eg, EHDEN project) learning and experience including processes and documentation for the betterment of real‐world health research.^60^

## 6 CONCLUSIONS

### 6.1 Lessons learned

Most partners agreed that being part of a highly interdisciplinary consortium like EMIF created tremendous value, as this facilitated forming cross‐topic collaborations, which allowed to perform complex research projects. As an example, a combined EMIF‐AD and EMIF‐MET study investigated (potentially AD‐related) cerebrospinal fluid changes in insulin‐resistant men and could only be achieved through a close interaction between these teams in a common environment. The pre‐competitive spirit of this collaboration also allowed researchers to share, access and reuse the data which had been challenging to do before EMIF. Furthermore, the project emphasized the importance of meticulous but flexible planning while designing the structure, especially for the interdisciplinary projects. While most project partners found the interdisciplinary structure to be beneficial, some partners did indicate that the sheer complexity associated with this structure led to challenges in comprehending the project structure and staying up to date of all project developments. This reinforces the need for excellent project coordination and internal communication. Therefore, it was strongly recommended to carefully discuss the project structure and management beforehand, and to keep the final outcome in mind while executing smaller tasks, all while making sure that all stakeholders are engaged throughout the project. Further, as the project involved partners from multiple disciplines, it was essential to understand the individual perspectives, assumptions and expectations while working together to achieve a common goal. Additionally, given the dynamic nature of pharmaceutical industry, more overall flexibility can be offered in the IMI projects to better allow partners to fulfil their commitments. Taken together, the most highly valued aspect of EMIF was the large and interdisciplinary structure, which allowed partners to gain many new insights, learning and experiences via collaborating with the other partners, effectively enlarging the network. Therefore, the EMIF project can be considered a one of its kind project, for which the expertise and experiences gained will be beneficial for all projects in the field of real‐world evidence.

### 6.2 Future opportunities

Because of the many positive experiences, it is no surprise that nearly all of the project partners indicated that they would like to take part in future IMI projects. Besides these future IMI projects, partners were also interested in further developing the EMIF outcomes and achievements. For potential new IMI projects, the partners suggested themes that were patient‐centric, with focus on outcomes, data sharing and with an aim to better understand the disease. Since the execution of these projects will require collection and processing of data, it is only logical that the data within EMIF will be reused for future research. In line with this, as discussed above, the EMIF catalogue continues to be operational and provides data for the projects within and outside EMIF. Since EMIF was developed as a sustainable model, the solutions provided within EMIF such as the catalogue, tranSMART, cohort explorer, deliverables, documents (eg, protocols) and so forth can and should be leveraged to future projects. To this effect, some of the participating partners have already incorporated parts of these solutions in their institution. Also, the newly established collaborations developed in EMIF can be carried forward by the partners to further their research and this is turn, will also ensure sustainability of EMIF model.

## CONFLICT OF INTEREST

The authors declare no conflict of interest in the publication of this manuscript.
